# Taxonomy of Atlantic Central African orchids 5. A new species of *Angraecum* sect. *Conchoglossum* (Orchidaceae, Angraecinae) from Gabon and Cameroon

**DOI:** 10.3897/phytokeys.61.7017

**Published:** 2016-02-25

**Authors:** Vladimir Ječmenica, Vincent Droissart, Nausicaa Noret, Tariq Stévart

**Affiliations:** 1 Laboratoire d’Écologie végétale et Biogéochimie, CP 244, Université Libre de Bruxelles, Boulevard du Triomphe, B-1050 Brussels, Belgium; 2 Herbarium et Bibliothèque de Botanique africaine, CP 265, Université Libre de Bruxelles, Boulevard du Triomphe, B-1050 Brussels, Belgium; 3 Institut de Recherche pour le Développement (IRD), Unité Mixte de Recherche AMAP (Botanique et Bioinformatique de l’Architecture des Plantes), Boulevard de la Lironde, TA A-51/PS2, F-34398 Montpellier Cedex 5, France; 4 Missouri Botanical Garden (MBG), Africa & Madagascar Department, P.O. Box 299, St. Louis, Missouri 63166-0299, U.S.A.; 5 Plant Systematic and Ecology Laboratory, Higher Teacher’s Training College, University of Yaoundé I, Yaoundé, Cameroon; 6 Botanic Garden, Meise, Domein van Bouchout, Nieuwelaan 38, B-1860 Meise, Belgium

**Keywords:** Angraecoid, Campo-Ma’an National Park, Ivindo National Park, Monts de Cristal National Park, IUCN Red List Categories and Criteria

## Abstract

Recent field inventories and taxonomic research in Central Africa have resulted in the discovery of many new orchid species. Five specimens of an apparently new *Angraecum* species were collected in Gabon and Cameroon. They stand out for their hanging habit and short zig-zag stem. Morphology of leaves and habit is somewhat comparable to *Angraecum cultriforme* and *Angraecum stolzii*, two species from East Africa. Flowers of the novelty share the general morphology of *Angraecum pyriforme* from which the new species is distinguished by being smaller and with a different lip-spur ratio. Here we show that these five specimens represent a new species, described here as *Angraecum lanceolatum*. The distinguishing traits include thin lanceolate leaves, convolute distally, with a rhombic lip shape. Dichotomous key to four Central African species of sect. *Conchoglossum* and a table of the diagnostic characters of the seven related Continental African *Angraecum* taxa are included here. A preliminary assessment of the conservation status of *Angraecum lanceolatum* is provided, using the IUCN Red List Categories and Criteria.

## Abstract (fr)

Des travaux récents d’inventaires et de taxonomie ont abouti à la découverte de nombreuses espèces nouvelles d’orchidées en Afrique centrale. Cinq spécimens n’appartenant à aucune espèce d’*Angraecum* connue à ce jour ont été récoltés au Gabon et au Cameroun. Ces spécimens se distinguent par un port pendant et une tige courte disposée en zigzag. Leur morphologie foliaire et leur port sont proches de ceuxd’*Angraecum cultriforme* et d’*Angraecum stolzii*, deux espèces d’Afrique de l’Est. Les fleurs de cette nouveauté taxonomique présentent la morphologie générale d’*Angraecum piriforme*, mais sont cependant plus petites et présentent un rapport différent de la longueur du labelle sur celle de l’éperon. Nous montrons ici que ces cinq échantillons représentent une nouvelle espèce, décrite sous le nom d’*Angraecum lanceolatum*. Les traits distinctifs de ce nouveau taxonsont ses feuilles minces, lancéolées et tordues à l’apex, ainsi que la forme du labelle en losange. Une clé dichotomique des quatre espèces de la section *Conchoglossum* présentes en Afrique centrale et une table des caractères diagnostiques des six taxons proches d’*Angraecum lanceolatum* présents en Afrique continentale sont proposées. Le statut de conservation d’*Angraecum lanceolatum* est évalué selon la méthodologie de la Liste Rouge de l’UICN.

## Introduction

According to the latest count of WCSP (Govaerts et al. 2015), the genus *Angraecum* Bory comprises 223 species. With 173 species recorded in the Malagasy region (Govaerts et al. 2015), Madagascar and the Mascarenes are considered as the centre of diversity of *Angraecum*. Nevertheless, Central Africa also shows a high orchid diversity and endemism rate (Stévart 2003, Droissart 2009) where many new species remain to be described. A cultivation system established in São Tomé, Gabon, Equatorial Guinea and Cameroon by Stévart (2003) and his collaborators has allowed collection of thousands of flowering specimens. This has enabled taxonomic revisions of several orchid genera (Verlynde et al. 2013, Simo-Droissart et al. 2014) and the description of more than 25 new orchid taxa (e.g. Droissart et al. 2014, Stévart et al. 2014, D’Haijere et al. 2015), many of which still remain to be published.

A revision of *Angraecum* species belonging to sections *Afrangraecum* Summerh. and *Conchoglossum* Schltr. was conducted by the first author in 2015. A careful examination of specimens from main herbaria has confirmed the status of five new species, of which one is described here.

The first collection of the new species originates from Mont Seni in the Monts de Cristal National Park in Gabon (IUCN Category II National Park). This specimen was collected by Nguema Miyono (*N. Miyono 2037*) in 2001 and deposited in BRLU and LBV (abbreviations after Thiers continuously updated). Unfortunately, the material was sterile and identified as *Angraecum angustipetalum* Rendle. A few years later, during fieldwork in the Ivindo National Park in Gabon, a living plant of the same species was collected by Diosdado Nguema. The specimen was sent to the garden of M. Biteau (Jardi-Gab, Libreville) who cultivated it in his shade-house under number *BTO23*. Since then, the plant has produced three flowering specimens (*D. Nguema s.n.*, *JBB 244* and *JBB 263*) after which it died. Following examination of the three flowering specimens and the living plant (*BTO23*), Stévart considered it as a potentially new species. Finally, fieldwork conducted in Cameroon by Droissart in February 2015 enabled another collection of that new species (*Droissart* et al. *1874*). The specimen was collected in the Campo-Ma’an National Park (South Region of Cameroon) and cultivated in Yaoundé shade-house under number *Y 5652 NY* where it flowered in June 2015. Comparison of these five specimens with the type material of related *Angraecum* species confirmed that these specimens represent a new species, described here as *Angraecum lanceolatum*.

This paper is the fifth in a series of publications (Stévart et al. 2010, Droissart et al. 2014, Stévart et al. 2014, D’Haijere et al. 2015) based on recent intensive fieldwork and focusing on collections-based taxonomic revisions of Orchidaceae in Atlantic Central Africa.

## Material and methods

This study was conducted under the framework of the first author’s Master’s thesis. A revision including 109 specimens from all *Angraecum* species belonging to sections *Afrangraecum* and *Conchoglossum* was undertaken. Collections of BR, BRLU, K, WAG, MA, MO, P and YA were examined and did not reveal any additional specimens of the new species. Description of the new species is based on five spirit preserved specimens originating from Gabon and Cameroon. The terminology used for description followed Systematics Association Committee for Descriptive Biological Terminology (1962a, 1962b), *Botanical Latin* (Stearn 1992) and *The Kew Plant Glossary* (Beentje 2010). Two living specimens of the new species were collected by teams of the Missouri Botanical Garden (MBG) and the Institut de Recherche pour le Développement (IRD) during fieldwork in Gabon and Cameroon. Sterile material collected in the field was grown in the shade-houses until obtaining flowered specimens preserved as spirit collections. Colour and habit characteristics given are based on the field data and high resolution photographs. Additional photographs, measurements and morphological study of spirit material were carried out using an optic microscope Zeiss STEMI SV11.

A preliminary risk of extinction assessment was made using the IUCN Red List Categories and Criteria (IUCN 2001, 2014). Georeferenced specimen data were imported into GIS to calculate area of occupancy (AOO) and extent of occurrence (EOO). The cell size for AOO was set 2 × 2 km as recommended by IUCN (2014). Each locality was regarded as a separate subpopulation. The number of ‘locations’ (as defined by IUCN 2014) was calculated with regard to the kind of threats, such that a single ‘location’ may encompass more than one adjacent population.

## Taxonomic treatment

### Angraecum lanceolatum

Taxon classificationPlantaeAsparagalesOrchidaceae

Ječmenica, Stévart & Droissart sp. nov.

urn:lsid:ipni.org:names:77153391-1

Figs 1 , 2

#### Diagnosis.

*Angraecum lanceolatum* is close to *Angraecum stolzii* Schltr. (1915) but differs from it by shorter narrowly ovate leaves that convolute in the distal half, by a shorter zig-zag stem and by a rhombic lip shape. The species also resembles *Angraecum cultriforme* Summerh. (1958) but differs from it by smaller flower size and slightly curved spur. *Angraecum lanceolatum* is also close to *Angraecum pyriforme* Summerh. (1936) in the shape of floral parts, but differs from it by previously stated vegetative characters, hanging habit, single-flowered inflorescence and smaller flower.

#### Type.

Cameroon. South Region of Cameroon, Campo-Ma’an National Park, nearby villages of Ebianemeyong and Nyabissan, 02°29.2488'N, 010°19.9026'E, 14 Feb 2015, *V. Droissart, T. Couvreur & N. Kamdem 1874* (holotype: BRLU!; isotype: YA!).

#### Description.

Small epiphytic herbaceous plant. Stem hanging, slightly zig-zag in form, unbranched, up to 8.5 cm long. Leaves alternate, spaced, narrowly ovate to lanceolate, sometimes slightly falcate and always convolute in the distal part, margins entire; distinct midvein forming slight channel, accompanied with 2 or 3 nerves on each side merging into one throughout; small stomata spots visible in young leaves; leaf apex unequally bilobed, acuminate, with the larger lobe 1.5–2.8 mm long and the smaller 0.3–1.2 mm long, leaf blade 2.3–4.1 × 0.6–0.9 cm; leaf internode about 5–6 mm long. Inflorescence single flowered, eventually two-flowered; peduncle elongated 13–23 mm long, opposite to the leaf at the node. Bracts acute, 2 mm long. Flowers white, opening diameter about 12.5 mm. Ovary and pedicel not resupinate, 8 mm long. Dorsal sepal 6.2–8.5 × 3 mm, elliptic, acute, thick, with entire margins. Lateral sepals 6–7 × 2–2.2 mm, elliptic, acute, thick, with entire margins. Petals 5–6.5 × 2–2.2 mm, obliquely elliptic, acute, entire margins, similar in shape to lateral sepals. Lip 5–6 × 4.5–5 mm, concave, rhombic when flattened, widest between first third and the half, acute; spur 16–19.5 mm, cylindric, slender, straight, somewhat elliptically inflated and greenish at the apex. Column 1.5 × 2 mm. Pollinia 2, pyriforme. Fruit capsule, 18–24 × 3.5–5 mm.

**Figure 1.**
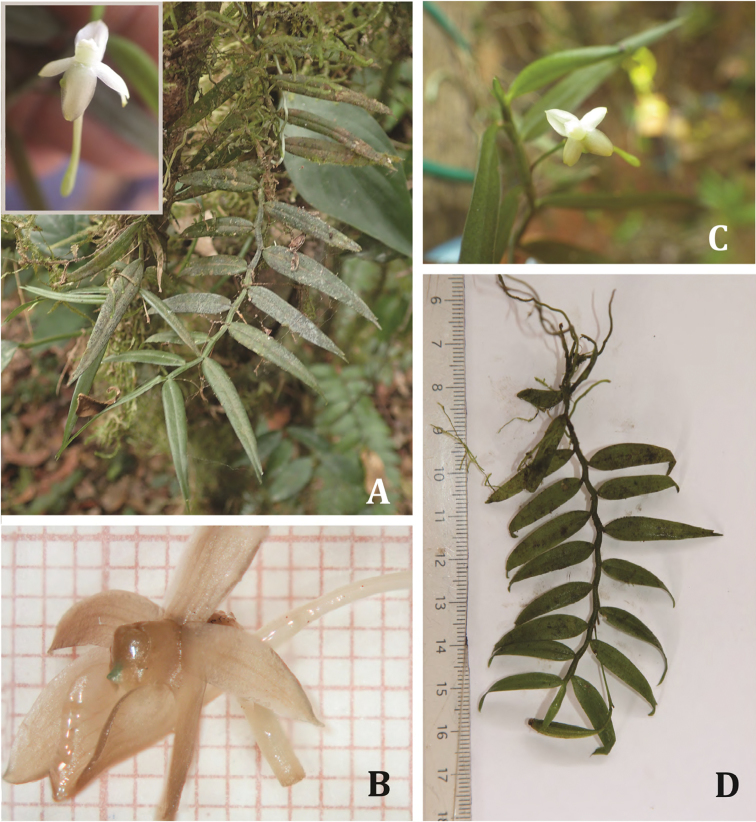
Photographs of living specimen of *Angraecum lanceolatum* (**A, C, D** *V. Droissart* et al. *1874* **B** *J.P. Biteau 263*): **A** habit and top view of the flower **B** half front view of the flower (from spirit material) **C** inflorescence and flower **D** habit and peduncle with fruit. Photographs taken by: **A, D** V. Droissart; **B** V. Ječmenica; **C** G. Kamdem.

**Figure 2.**
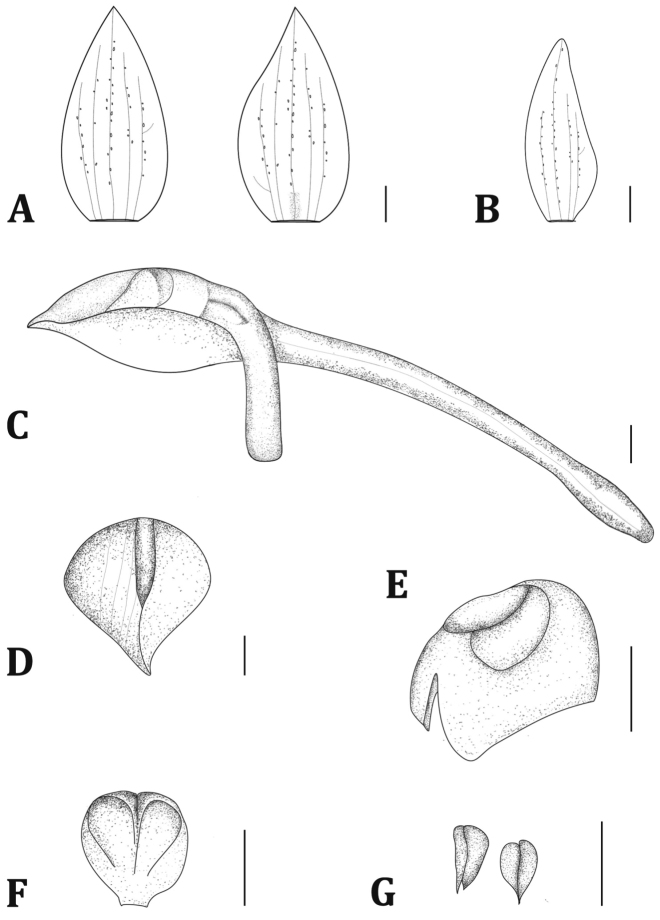
*Angraecum lanceolatum*: **A** Sepals **B** Petal **C** Lip, column, ovary and pedicel, spur **D** Lip, flattened, overhead view **E** Column without anther cap **F** Anther cap **G** Pollinia. Bars represent 1 mm. Illustration of specimen *D. Nguema s.n.* by Danka Ječmenica and Vladimir Ječmenica.

#### Additional specimens.

Gabon. Monts de Cristal National Park. Mont Seni, 13 Sept 2001, *Nguema Miyono 2037* (LBV, BRLU!); Ivindo National Park, near Langoué Bai, 17 Sept 2005, *D. Nguema s.n.* (BRLU!); ibid., *J.P. Biteau 263* (BRLU!); ibid., *J.P. Biteau 244* (BRLU!).

#### Distribution and habitat.

**Figure 3.**
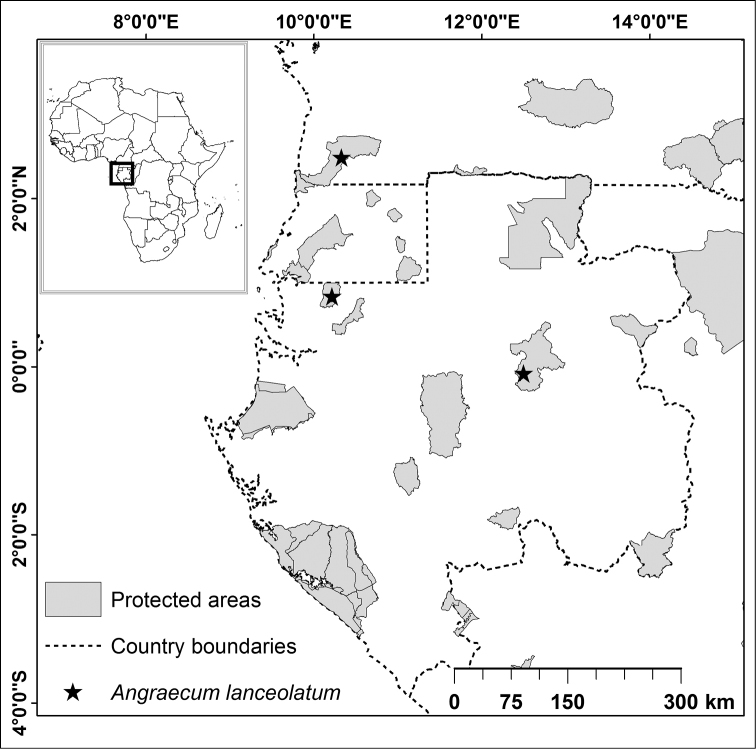
Distribution of *Angraecum lanceolatum* in tropical Africa.

Endemic to the Lower Guinea Domain (Cameroon and Gabon, Fig. 3). The specimen collected in Cameroon was found at 850 m elevation in submontane forest with *Gilbertiodendron unijugum* (Pellegr.) J. Léonard (Fabaceae). The plant was epiphyte at about 1.5 m from the ground, on the trunk of a shrub with a diameter less than 10 cm.

#### Phenology.

Flowering occurs in June and September.

#### Conservation.

IUCN Red List category: Least Concern [LC]. The extent of occurrence (EOO) of *Angraecum lanceolatum* is estimated to be over 23,884 km^2^, exceeding the 20,000 km^2^ upper limit for Vulnerable status under the criterion B1, whereas its area of occupancy (AOO) is estimated to be 12 km^2^ (which falls within the limits for Endangered status under the criterion B2). The species is now known from three subpopulations in Gabon and Cameroon. These three subpopulations represent three different locations (sensu IUCN 2014), less than five locations, which is the upper limit for Endangered status under the subcriterion ‘a’ of criterion B2. *Angraecum lanceolatum* has only been collected in protected areas (Monts de Cristal and Ivindo National Parks in Gabon and Campo-Ma’an National Park in Cameroon). None of these protected areas is under threat and they appear well managed. *Angraecum lanceolatum* is thus not threatened. The available information suggests that the number of subpopulations and mature individuals, as well as its EOO and AOO, will not decrease noticeably in 10 years or 3 generations the future. Application of the IUCN criteria therefore indicates that it cannot be regarded as Endangered despite the fact that its AOO is limited. *Angraecum lanceolatum* is therefore assigned a preliminary status of LC.

#### Etymology.

The specific epithet of the new species owes to the particular leaf shape. Even though there are several interpretations of “lanceolate” shape according to different authors (Linnaeus, Lindley), we relied on the current depiction from Beentje (2010) that describes it as narrowly ovate and tapering to a point at the apex.

### Taxonomic key to section *Conchoglossum* species from Central Africa

**Table:** 

1a	Leaves oblong; spur sigmoid, apex not or rarely slightly cylindrically inflated	***Angraecum moandense***
1b	Leaves not oblong; spur straight or slightly curved, apex inflated	**2**
2a	Leaves broadly ovate; spur apex circularly inflated	***Angraecum egertonii***
2b	Leaves narrowly ovate, lanceolate or elliptic; spur elliptically inflated	**3**
3a	Leaves very fleshy, broadly elliptic, up to 2.7 cm long; lip elliptic to ovate	***Angraecum lisowskianum***
3b	Leaves thin, narrowly ovate to lanceolate, distally convolute up to 4.1 cm long; lip rhombic	***Angraecum lanceolatum***

### Notes

The diagnostic characters of species from Central African region that belong to the section *Conchoglossum*, as well as one morphologically related species of the section *Afrangraecum* are summarized (Table 1). Morphometric results of the mentioned Master’s thesis and molecular data on *Angraecum* (Stévart unpublished) confirmed the status of the new species. Vegetative morphology and habit of the new species resemble *Angraecum stolzii* Schltr. in having single flowered inflorescence and sometimes slightly falcate leaves. Large and small leaf apex lobes are not prominent as in *Angraecum stolzii*, in which the larger lobe reaches at least 10 mm, comparing to a maximum of 2.5 mm in *Angraecum lanceolatum*. Spur is similar in shape but in the new species it is at least three times longer than the lip, while the flower in *Angraecum stolzii* has approximately equal spur and lip lengths.

**Table 1.** Morphological comparison of characters for seven related continental African *Angraecum* species. All species belong to section *Conchoglossum* except for *Angraecum pyriforme*, which is a member of section *Afrangraecum*. Diagnostic characters are indicated in bold.

Taxa	Distribution	Stem size	Leaves	Peduncle	Sepals	Petals	Lip	Spur
*Angraecum stolzii* Schltr.	Democratic Republic of the Congo, Tanzania, Malawi, Zambia	15.5–40 cm	5.6–8 × 0.5–0.9 cm **linearly falcate**, acuminate apex	1.3–2.2 cm	Elliptic, **acuminate, lateral falcate**, 4–7.3 mm × 1.8 mm	Elliptic, acuminate, **3–6.2 × 1.3 mm**	**Ovate**, acute, **3–5.5 × 2–2.5 mm**	Straight or slightly curved, elliptic apical inflation, **2.5–4.6 mm long**
*Angraecum egertonii* Schltr.	Nigeria, Cameroon, Gabon	10–22cm	2.7–4 × 1.5–1.9 cm **ovate**, acute apex	2–4 cm	Elliptic, **acute, lateral sometimes falcate**, 8–12.8 × 2–4 mm	**Elliptic to falcate**, acute, 7.5–11.5 × 1.5–2.5 mm	Elliptic to ovate, acute, 7–11 × 2.5–4.5 mm	Bent upwards with **circular apical inflation**, 6.5–8.5 mm long
*Angraecum pyriforme* Summerh.	Ivory Coast, Nigeria	8–11 cm	7–11 × 1–2.2 cm narrowly elliptic, **obliquely round apex**	2–4 cm	Elliptic, **acute, 7–11 × 2.5–4 mm**	**Obliquely elliptic**, acute, **6.5–8.5 × 1.5–4 mm**	**Rhombic**, **acuminate**, 6–7.5 × 4–4.5 mm	Straight with elliptic apical inflation, **10.5–15 mm long**
*Angraecum lisowskianum* Szlach. & Olsz.	Nigeria, Cameroon, Equatorial Guinea	7.5–11 cm	1.7–2.65 × 0.7–1.1 cm elliptic, **subacute apex**	0.7–1.8 cm	Elliptic, **acute, lateral subfalcate**, 6–10.5 × 1.5–3 mm	Elliptic, acute 7.5–9.5 × 1–2.2 mm	Elliptic to ovate, acuminate 6.5–8.5 × 4 mm	Straight with elliptic apical inflation, **15–21.5 mm long**
*Angraecum cultriforme* Summerh.	Kenya, Tanzania, Malawi, Mozambic, Zambia, Zimbabwe, KwaZulu-Natal	8–15 cm	3.7–6 × 0.4–0.8 cm **elliptic to linearly falcate**, acute apex	1.5–3 cm	Elliptic, **acuminate**, **12.5–18 × 2.3–3 mm**	Elliptic, acuminate, **11–15 × 2–2.5 mm**	**Ovate**, acuminate, **10–14 × 6 mm**	Straight, **slightly ascending** with elliptic apical inflation, **20–26 mm long**
*Angraecum lanceolatum*	Cameroon, Gabon	Up to 8.5 cm	2.3–4.1 × 0.6–0.9 cm **narrowly ovate to lanceolate**, acuminate apex	1.3–2.3 cm	Elliptic, **acute, 6–8.5 × 2–3 mm**	**Obliquely elliptic**, acute, **5–6.5 × 2–2.2 mm**	**Rhombic**, **acute**, **5–6 × 4.5–5 mm**	Irregularly straight with elliptic apical inflation, **16–19.5 mm**
*Angraecum moandense* De Wild.	Ghana, Republic of Guinea, Ivory Coast, Liberia, Nigeria, Togo, Central African Republic, Cameroon, Republic of the Congo, Gabon, Gulf of Guinea Islands, Rwanda, Democratic Republic of the Congo, Tanzania, Uganda	6–15 cm	4.2–9.2 × 0.7–1 cm **oblong**, round apex	0.6–3.5 cm	Elliptic, **acuminate**, 8–15.5 × 2–3.5 mm	Obliquely linear to elliptic, **acuminate, 8–14 × 1–2.5 mm**	Elliptic to slightly ovate, **acuminate to cuspidate**, 8–11.5 × 2.5–3.5 mm	**S-shaped** with occasionally slightly cylindrically inflated apex, 14–26 mm long

Floral morphology, particularly the lip shape of new species is similar to *Angraecum pyriforme* Summerh. from the sect. *Afrangraecum*. Nevertheless, ratio between lip and spur lengths is close to 1:3 in the new species compared to 1:2 in *Angraecum pyriforme*. Additionally, the new species has a distinctive habit.

The novelty is a representative of *Conchoglossum* section according to Stewart et al. (2006, see also Summerhayes 1958, *Angraecoides* sensu Garay 1973), due to its continental distribution and its white single flowered inflorescence.

## Supplementary Material

XML Treatment for Angraecum lanceolatum
